# Promoter hypermethylation of *MGMT*, *CDH1, RAR-β* and *SYK* tumour suppressor genes in granulosa cell tumours (GCTs) of ovarian origin

**DOI:** 10.1038/sj.bjc.6601567

**Published:** 2004-02-17

**Authors:** V S Dhillon, A R Young, S A Husain, M Aslam

**Affiliations:** 1 Department of Molecular and Clinical Genetics, Royal Prince Alfred Hospital, Camperdown NSW 2050, Australia; 2 Cytogenetics Laboratory, Department of Biosciences, Jamia Milia Islamia, New Delhi 110 025, India

**Keywords:** ovarian carcinoma, hypermethylation of *MGMT*, CDH1, *RAR-β* and *SYK* gene

## Abstract

Ovarian carcinoma (OC) is a leading cause of death among women throughout the world. A number of cancer-associated genes have been shown to be inactivated by hypermethylation of CpG islands during tumorigenesis. We tested the hypothesis that methylation status of *MGMT*, *CDH1*, *RAR-β* and *SYK* could be important in the ovarian tumorigenic process and can lead to the gene(s) inactivation. Therefore, we assessed the promoter hypermethylation of *MGMT*, *CDH1*, *RAR-β* and *SYK* in 43 ovarian granulosa cell tumours (GCTs) (adult type) using methylation-specific PCR. These tumours are relatively rare, accounting for approximately 3% of all ovarian cancers. Hypermethylation of *MGMT* (in 14 tumours), *CDH1* (in nine tumours), *RAR-β* (in eight tumours) and *SYK* (in seven tumours) have been found. Selective loss of *RAR-β* and *RAR-β2* mRNA has been found in seven patients, while that of *MGMT* and *SYK* in three patients who also show aberrant methylation in promoter region of *RAR-β* in addition to *MGMT*, *SYK* and *CDH1* genes. Promoter CpG hypermethylation may be an alternative to mutation(s) to inactivate tumour suppressor genes such as *MGMT*, *CDH1*, *RAR-β* and *SYK*, and this can also be an early event in the pathogenesis of OCs. Moreover, hypermethylation of the *MGMT* and *CDH1*, *MGMT* and *RAR-β* and *CDH1* and *RAR-β* promoters occurred concordantly (*P*< 0.001, 0.0421 and 0.0005 respectively; Fischer's exact test). In addition to this, monosomy 22 and trisomy 14 have also been found in 10 tumours. It is clear from the results that hypermethylation of the promoter region of these tumour suppressor genes, monosomy 22 and trisomy 14, may be critical steps in the tumorigenesis, which consequently play a permissive role for tumour aggressiveness. All these events might play an important role in the early clinical diagnosis of the disease. Our results, therefore, suggest a potential role for epigenetic modification of these critical tumour suppressor genes in pathways relevant to the transformation and differentiation of rare type of ovarian cancer (GCTs).

Ovarian tumours are relatively common, and are the most lethal tumour of the female genital tract (Evan *et al*, 1999). Among ovarian cancers, granulosa cell tumours (GCTs) are relatively rare, accounting for approximately 3% of all ovarian cancers. The aetiology of these GCTs remains unclear. It is now accepted that accumulation of multiple genetic aberrations can lead to the development of most carcinomas. DNA replication errors (RER) have been detected in epithelial ovarian cancer, as well as in other human cancer types, and a DNA mismatch repair deficiency may be involved in their development and or progression (Suzuki *et al*, 2000). Genetic or epigenetic alterations in a variety of genes are fundamental to the processes of growth, cell proliferation, differentiation and programmed cell death and removal. Each alteration may be mediated through gross chromosomal changes and, hence, has the potential to be detected cytogenetically (Sandberg, 1991). It is generally thought that the events causing the activation of certain oncogenes and/or inactivation of tumour suppressor genes lead to tumour development and progression, which is caused by genetic alterations such as chromosome deletions and or loss of function mutations in the coding regions (in CpG sites) of the tumour suppressor genes.

*O*^*6*^*-methylguanine-DNA methyltransferase* (*MGMT*; 10q26; GenBank Accession No. U95038) is a DNA repair gene, which removes methyl groups as well as larger adducts at O^6^ of guanine. The alkylation of DNA at O^6^ position of guanine is associated with the formation of DNA mutations in cancers. CpG islands within −249 to +259 relative to transcriptional start site were chosen to study the role of promoter methylation on gene silencing. Methylation of *MGMT* gene has been reported in various carcinomas such as gliomas, non-small-cell lung carcinoma, lymphoma, head and neck carcinoma and colorectal cancers (Esteller *et al*, 1999b, 2000; Rosas *et al*, 2001). *E-cadherin* is an *M*_r_ 120 000 transmembrane glycoprotein (16q22.1; GenBank Accession No. D49685) expressed on the surface of epithelial cells and is essential for the maintenance of normal tissue homeostasis and architecture (Takeichi, 1995). Reduced *E-cadherin* expression has been associated with an unfavourable prognosis of cancers originating from breast, lung, nasopharynx, bladder and gastric mucosa (Zheng *et al*, 1999; Garcia del Muro *et al*, 2000; Heimann *et al*, 2000), and the important mechanism for loss of expression is methylation of the 5′ CpG islands within the promoter, which results in transcriptional repression of the gene (Yoshiura *et al*, 1995). CpG island in exon 1 of E-cadherin gene (−126 to +144) relative to transcription start site was selected to detect the promoter methylation effects on gene silencing. Retinoic acid receptor beta (*RAR-β*;3p24.3-24.2) induces local chromatin changes at levels of target genes containing retinoic acid responsive elements (*RAREs*) by recruiting multiprotein complexes with histone acetyltransferase (HAT) and histone deacetylase (HDAC) activities that dynamically alter chromatin structure and regulate gene expression (Chambon, 1996). A RARE is also located in the *RAR-β* promoter region and mediates *RAR-β* induction in response to RA in several cell lines and tissues (de The *et al*, 1990). Auto regulation of the *RAR-β* gene may play a critical role in amplifying the RA response. Lack of *RAR-β* expression has been observed in a number of malignant tumours, including carcinomas of the lung (Gebert *et al*, 1991), head and neck (Lotan *et al*, 1995) and breast (Seewaldt *et al*, 1995). The overexpression of RAR-*β* induced growth arrest and apoptosis in oral cancer cell lines, and could function as a tumour suppressor (Hayashi *et al*, 2001). For *RAR-β* gene, methylation primers were designed as per the gene sequence (position 773–1007; transcription start site at position 844) to investigate the role of promoter hypermethylation in gene silencing. *SYK* gene (9q22; GenBank Accession No. Z29630) encodes a protein tyrosine kinase, Syk, which is highly expressed in haematopoietic cell, especially in T-cell and B-cell development and activation (Chu *et al*, 1998). Loss of Syk expression seems to be associated with malignant phenotype, such as increased motility and invasion (Coopman *et al*, 2000). Exon 1 (107 bp) and exon 2 (448 bp) are separated by ∼4.1 kb intron and the transcription start site is located in exon 2. CpG island spans from 350 bp 5′ of exon 1 to 150 bp of intron 1. A 600 bp region around exon 1 contains 62 CpG sites. Therefore, the high density of CpG sites in the 5′-regulatory region suggested that promoter methylation may influence the transcriptional regulation of *SYK* expression. Methylation-specific primers were chosen to cover nine CpG numbered 17–21 (forward) and 47–50 (reverse), whereas unmethylation-specific primers covered eight CpGs numbered 18–22 (forward) and 35–37 (reverse). Methylation of CpG sites in the promoter region has become recognised as an alternative mechanism for the inactivation of certain tumour suppressor genes, which is thought to be the most common mechanism of gene inactivation in various tumours.

Therefore, all these above findings led us to investigate the role of promoter hypermethylation of *MGMT*, *CDH1*, *RAR-β* and *SYK* genes, and mRNA expression of *RAR-β* and *RAR-β2* genes in GCTs of ovaries.

## MATERIAL AND METHODS

The subjects were 43 patients affected with GCTs of ovaries without a positive family history. All of them were untreated at the time of the study. The tumour grading is as follows: 19 FIGO stage IA, 10 FIGO stage IB and 14 FIGO stage IC. All these diagnoses were reviewed by a gynaecologic pathologist, and the tumours were assessed using standard criteria (Russel and Bannatyne, 1989). An informed consent was taken from all the subjects prior to the study. This study was approved by the Health Research Ethics Board of the Faculty of Natural Sciences. In all, 25 G-banded metaphases were obtained after a 5-day-old culture of an overnight collagenase disaggregated specimen of the tumour.

### Methylation-Specific PCR (MSP)

DNA methylation patterns in CpG islands of tumour suppressor genes *MGMT*, *CDH1*, *RAR-β* and *SYK* were determined by chemical modification with sodium bisulphite. Briefly, 1 *μ*g DNA was denatured by NaOH (50 *μ*l, final concentration, 0.2 M) for 10 min at 37°C; 1 *μ*g of salmon sperm DNA (Sigma) was added as carrier before modification. Freshly prepared 30 *μ*l of hydroquinone (10 mM, Sigma) and 520 *μ*l of sodium bisulphite (3 M, pH 5.0, Sigma) were mixed and the samples were incubated under mineral oil at 55°C for 16 h. The DNA samples were desalted through Wizard columns (Promega, Madison, WI, USA), then desulphonated by NaOH (final concentration, 0.3 M) treatment for 5 min at room temperature, followed by ethanol precipitation. DNA was resuspended in water and used immediately or stored at −20°C. PCR primers that distinguish between these methylated and unmethylated DNA sequences were used. Primer sequences of all genes for both the methylated and the unmethylated form, annealing temperatures and the expected PCR product sizes are summarised in Table 1

**Table 1:** Summary of primer sequences, annealing temperature and PCR product sizes used for MSP

**S. no.**	**Name and type**	**Primer sequence**	**PCR conditions**	**Cycles**	**bp**
1	MGMT-Met: F	5′-TTT CGA CGT TCG TAG GTT TTC GC-3′	95°C × 30s; 61°C × 45s; 72°C × 45 s	35	81
	R	5′-GCA CTC TTC CGA AAA CGA AAC G-3′			
2	MGMT-UnM: F	5′-TTT GTG TTT TGA TGT TTG TAG GTT TTT GT-3′	95°C × 30s; 61°C × 45s; 72°C × 45 s	35	93
	R	5′-AAC TCC ACA CTC TTC CAA AAA CAA AAC A-3′			
3	CDH1-Met: F	5′-TGT AGT TAC GTA TTT ATT TTT AGT GGC GTC-3′	95°C × 30s; 64°C × 30s; 72°C × 30 s	40	112
	R	5′-CGA ATA CGA TCG AAT CGA ACC G-3′			
4	CDH1-UnM: F	5′-TGG TTG TAG TTA TGT ATT TAT TTT TAG TGG CGT C-3′	95°C × 30s; 62°C × 30s; 72°C × 30 s	40	120
	R	5′-ACA CCA AAT ACA ATC AAA TCA AAC CAA A-3′			
5	RAR*β*-Met: F	5′-GGT TAG TAG TTC GGG TAG GTT TTA TC-3′	95°C × 30s; 58°C × 30s; 72°C × 30 s	35	235
	R	5′-CCG AAT CCT ACC CCG ACG-3′			
6	RAR*β*-UnM: F	5′-TTA GTA GTT TGG GTA GGG TTT ATT-3′	95°C × 30s; 58°C × 30s; 72°C × 30 s	35	233
	R	5′-CCA AAT CCT ACC CCA ACA-3′			
7	SYK-Met: F	5′-CGA TTT CGC GGG TTT CGT TC-3′	95°C × 30s; 67°C × 30s; 72°C × 30 s	35	243
	R	5′-AAA ACG AAC GCA ACG CGA AAC-3′			
8	SYK-UnM: F	5′-ATT TTT GTG GGT TTT GTT TGG TG-3′	95°C × 30s; 67°C × 30s; 72°C × 30 s	35	140
	R	5′-ACT TCC TTA ACA CAC CCA AAC-3′			

. For PCR amplification, 2 *μ*l of bisulphite-modified DNA was added in a final volume of 25 *μ*l of PCR mixture containing 1 × PCR buffer, MgCl_2_, deoxynucleotide triphosphates, and primers (100 pmol each per reaction) and 1 U of AmpiTaq Gold (Applied Biosystems, NJ, USA). Amplification was carried out in a 9700 Perkin–Elmer thermal cycler under the following conditions: 95°C for 10 min; 35 cycles of 95°C for 45 s, the specific annealing temperature for each gene for 1 min, and 72°C for 60 s; followed by a final 10 min extension at 72°C. Following sequencing, the primer pair was used to amplify bisulphite-modified DNA-containing gene for *E-cadherin* : (forward) 5′-GTT TAG TTT TGG GGA GGG GTT-3′ and (reverse) 5′-ACT ACT ACT CCA AAA ACC CAT AAC TAA-3′. The PCR cycle consisted of initial denaturation at 95°C for 5 min, 30 cycles (95°C × 30 s, 50°C × 30 s and 72°C × 30 s) followed by a final extension at 72°C for 5 min. The PCR product was then diluted 1 : 50 with sterile distilled water and 2 *μ*l of this 1 : 50 diluted product was then used for nested PCR using methylated and unmethylated primer pairs. Similarly, the following sequencing primer pair was used to amplify bisulphite-modified DNA-containing gene for *SYK*: (forward) 5′-GAT TAA GAT ATA TTT TAG GGA ATA TG-3′ and (reverse) 5′-CAC CTA TAT TTT ATT CAC ATA ATT TC-3′. The PCR cycle consisted of hot start at 95°C × 5 min and 35 cycles (95°C × 45 s, 58°C × 50 s and 72°C × 45 s) followed by a final extension at 72°C for 5 min. The PCR product was then diluted 1 : 50 with sterile distilled water and 2 *μ*l of this 1 : 50 diluted product was then used for nested PCR using methylated and unmethylated primer pairs. Each PCR product (10 *μ*l) was directly loaded onto 6% nondenaturing polyacrylamide gels, stained with ethidium bromide and directly visualised under UV illumination. The MSP for all samples was repeated twice to confirm their methylation status.

Statistical differences were assessed with Fisher's exact test by InStat 3 Windows software. Two-sided test were used to determine the significance. *P*-values less than 0.05 were regarded as statistically significant.

## RT–PCR

### RAR-β and RARβ-2

Total RNA was prepared using RNeasy mini-kit (Qiagen, CA, USA) as per the manufacturer's recommendations. In brief, total RNA was extracted from the specific cancerous tissues. Cell lysates were then homogenised by passing repeatedly through a 23-gauge needle followed by spinning through a QIAshredder (Qiagen, CA, USA). RNA concentrations were determined by measuring the absorbance (260 nm UV) using a spectrophotometer (Pharmacia). Total RNA (2 *μ*g) was used for the generation of cDNAs using Superscript reverse transcriptase (GIBCO BRL, Gaithersburg, MD, USA). The following primers were used to detect *RAR-β* and *RAR-β2* transcripts: *RAR-β* forward 5′-ACC AGC TCT GAG GAA CTC GTC CCA-3′ and *RAR-β* reverse 5′-AGG CGG CCT TCA GCA GGG TAA TTT-3′, and for *RAR-β2* forward (in exon 3), 5′-GCA TGG CAG AGT GCC CTA TC-3′; reverse (in exon 6), 5′-TCC CAG AGT CAT CCC TGC TTC AT-3′. PCR amplification was performed for 30 cycles at 95°C for 30 s, 60°C for 30 s (62°C for *RAR-β2*) and 72°C for 45 s (60 s for *RAR-β2)*. Human *GAPDH* was used as an internal control.

### MGMT gene

The following primer sequences were used for *MGMT* gene sense, 5′-CCTGGCT GAATGCCTATTTC-3′, and anti-sense, 5′-CAGCTTCCATAACACCTGTCTG-3′, which amplifies 116-bp PCR product. To verify the integrity of cDNA, *β2-microglobulin* expression was also analysed using the primer sequences sense, 5′-CATCCAGCGTACTCCAAAGA-3′, and *β2-microglobulin* anti-sense, 5′-GACAAGTCTGAATGCTCCAC-3′, which amplifies 165-bp PCR product. These two sets of primers span junctions between two exons, so amplification of the contaminating genomic DNA can be excluded. PCR amplification was performed for 94°C for 10 min, then 35 cycles of 94°C for 45 s, 57°C for 60 s and 72°C for 30 s, followed by a final elongation step at 72°C for 10 min.

### SYK gene

The following primer sequences were used for *SYK* gene sense, 5′-TGTCAAGGATAAGAACATCATAG-3′, and anti-sense, 5′-CACCACGTCAT AGTAGTAATTG-3′, which amplifies 507-bp PCR product. In this PCR, *β2-microglobulin* was also used as an internal control. The optimised PCR program was 94°C for 5 min, then 35 cycles of 94°C for 40 s, 62°C for 40 s and 72°C for 45 s, followed by a final elongation step at 72°C for 7 min.

The PCR products were subjected to electrophoresis in 2.0% agarose gels and visualised by ethidium bromide staining.

## RESULTS

Cytogenetic analysis performed on these tumours exhibited a 46,XX normal karyotype in 33 tumours. However, trisomy 14 and monosomy 22 were found in only 10 tumours (Table 2

**Table 2:** Promoter hypermethylation in different genes in ovarian cancer

			**Promoter hypermethylation**			**Expression of**						
**S. no.**	**Age (years)**	**Stage**	***MGMT***	***CDH1***	***RAR-β***	***SYK***	***RAR-β***	***RAR-β2***	***MGMT***	***SYK***	**Trisomy 14**	**Monosomy 22**
1	45	IA	+	+	+	−	−	−	+	+	Yes	Yes
2	55	IA	−	−	−	−	+	+	+	+	No	No
3	51	IA	−	−	+	+	+	+	+	+	No	No
4	65	IA	−	−	−	+	+	+	+	−	No	No
5	38	IA	+	−	−	−	+	+	+	+	Yes	Yes
6	36	IA	+	−	−	+	+	+	+	+	No	No
7	75	IA	−	−	+	−	+	−	+	+	Yes	Yes
8	81	IA	−	−	−	−	+	+	+	+	No	No
9	38	IA	−	−	−	−	+	+	+	+	No	No
10	47	IA	−	−	−	−	+	+	+	+	No	No
11	49	IA	−	−	−	−	+	+	+	+	No	No
12	64	IA	+	+	+	−	−	−	−	+	Yes	Yes
13	39	IA	−	−	−	−	+	+	+	+	No	No
14	42	IA	−	−	−	−	+	+	+	+	No	No
15	48	IA	+	+	+	−	−	−	+	+	Yes	Yes
16	53	IA	−	−	−	−	+	+	+	+	No	No
17	58	IA	−	−	−	−	+	+	+	+	No	No
18	61	IA	+	+	+	+	−	−	−	−	Yes	Yes
19	33	IA	−	−	−	−	+	+	+	+	No	No
20	52	IB	−	−	−	−	+	+	+	+	No	No
21	47	IB	−	−	−	−	+	+	+	+	No	No
22	51	IB	−	−	−	−	+	+	+	+	No	No
23	39	IB	−	−	−	−	+	+	+	+	No	No
24	49	IB	−	−	−	−	+	+	+	+	No	No
25	66	IB	+	+	+	+	−	−	−	−	Yes	Yes
26	62	IB	−	−	−	−	+	+	+	+	No	No
27	73	IB	+	+	+	−	−	−	+	+	Yes	Yes
28	58	IB	+	+	−	−	+	+	+	+	No	No
29	52	IB	−	−	−	−	+	+	+	+	No	No
30	55	IC	−	−	−	−	+	+	+	+	No	No
31	59	IC	−	−	−	−	+	+	+	+	No	No
32	62	IC	−	−	−	+	+	+	+	+	No	No
33	44	IC	+	−	−	+	+	+	+	+	Yes	Yes
34	42	IC	−	−	−	−	+	+	+	+	No	No
35	37	IC	+	−	−	−	+	+	+	+	No	No
36	66	IC	−	−	−	−	+	+	+	+	No	No
37	69	IC	+	−	−	−	+	+	+	+	No	No
38	61	IC	−	−	−	−	+	+	+	+	No	No
39	37	IC	−	−	−	−	+	+	+	+	No	No
40	45	IC	+	+	+	+	−	−	+	+	Yes	Yes
41	48	IC	−	−	−	−	+	+	+	+	No	No
42	71	IC	−	−	−	−	+	+	+	+	No	No
43	38	IC	+	+	−	−	+	+	+	+	No	No

+=hypermethylated; −=unmethylated; +=mRNA expression present; −: mRNA expression lost.

).

### MGMT, CDH1, RAR-β and SYK promoter hypermethylation

The methylation status of ovarian tumours (GCTs) was determined at the respective loci using MSP. DNA from 43 ovarian tumours was modified using sodium bisulphite, which converts all unmethylated cytosine residues to uracil, but leaves methylated cytosines unchanged. To confirm that the modification was successful, all samples were first amplified with primers specific for unmethylated DNA at the *p16* or *MLH1* loci (even tumours methylated at these loci would be expected to be positive due to contamination with normal tissue, which is known to be unmethylated). The samples were next subjected to MSP using primers specific for methylated DNA at the respective loci being studied.

**Figure 1:**
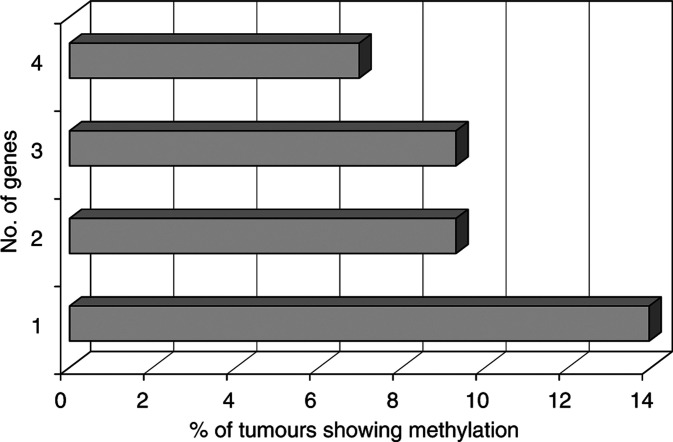
Percentage of distribution of the number of genes methylated in ovarian carcinoma.

**Figure 2:**
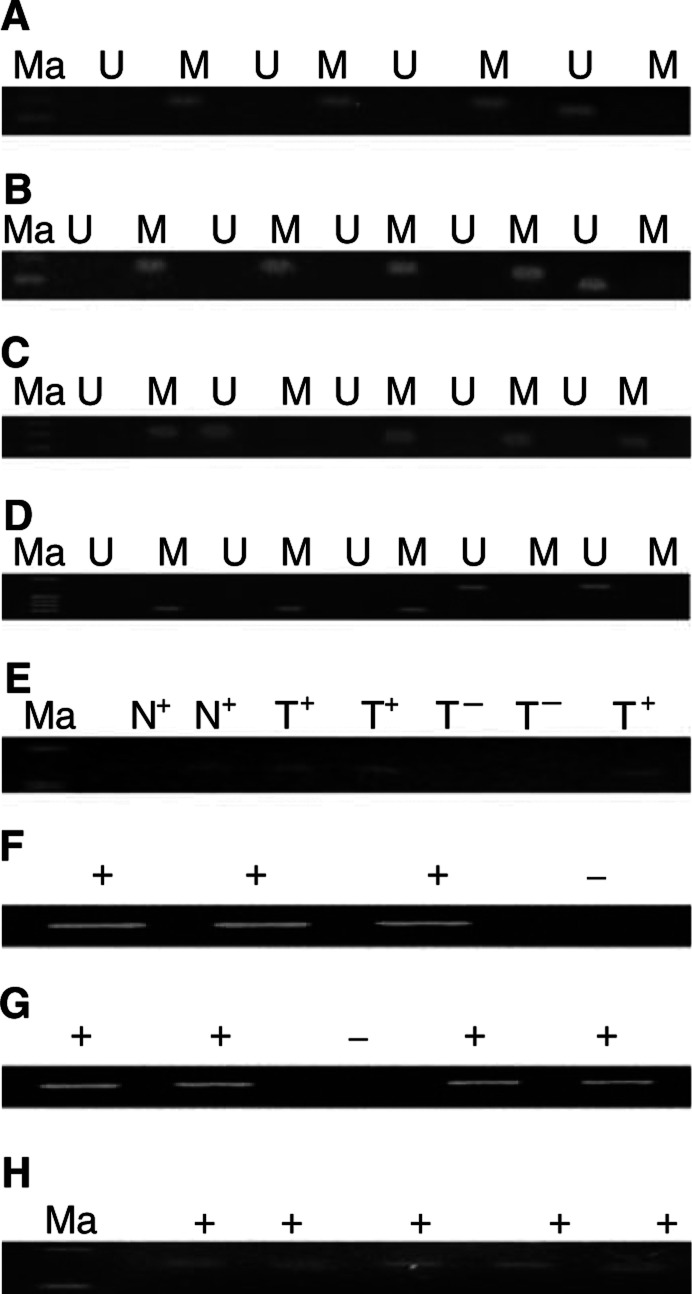
Representative examples of MSP analysis of *MGMT*, *CDH1*, *RAR-β* and *SYK* genes in GCTs of ovarian origin. (**A**) Unmethylated and methylated *MGMT* PCR products were detected in one and three cases. (**B**) Unmethylated and methylated *CDH1* PCR products were detected in one and four cases. (**C**) Unmethylated and methylated *RAR-β* products were detected in one and four cases. (**D**) Unmethylated and methylated *SYK* PCR products were detected in two and three cases, respectively. (**E**) Gene expression of *RAR-β* in GCTs of ovaries (**F**) loss of gene expression of *MGMT* gene in one patient (**G**) loss of gene expression of *SYK* gene in one patient and (**H**) GADPH as internal control (+) for expression studies: U, unmethylated PCR products; M, methylated PCR products; Ma, Molecular weight marker; N^+^, gene expression in lymphocytes; T^+^, gene expression in cancerous tissue; T^−^, loss of expression in cancerous tissues.

At least one of these four genes showed aberrant methylation in 17 tumour samples (17 out of 43). Methylation of only one gene was found in 13.95% (6 of 43) of tumours. The percentage of the tumours with methylation in genes 2, 3 and 4 is 9.3% (4 of 43), 9.3% (4 of 43) and 6.97% (3 of 43), respectively (Figure 1

). Three tumours were found to be hypermethylated for four tumour suppressor genes (Table 2) investigated in the present study. The representative examples of MSP are shown in Figure 2

.

We compared hypermethylation of *MGMT*, *CDH1*, *RAR-β* and *SYK* promoters in ovarian carcinoma (OC). As shown in Table 3

**Table 3:** Correlation of promoter methylation of *MGMT*, *CDH1*, *RARb* and *SYK* in ovarian carcinomas

	**Methylated**	**Unmethylated**	***P*-value**
**MGMT methylation status**			
*CDH1 methylation status*			
Methylated	9	0	0.001*
Unmethylated	5	11	

*RAR-β methylation status*			
Methylated	7	1	0.0421*
Unmethylated	7	10	

*SYK methylation status*			
Methylated	4	2	0.6609
Unmethylated	10	9	

***CDH1* methylation status**			
*RAR-β methylation status*			
Methylated	7	1	0.0005*
Unmethylated	2	15	

*SYK methylation status*			
Methylated	3	4	1
Unmethylated	6	11	

**RAR-β methylation status**			
*SYK methylation status*			
Methylated	2	5	1
Unmethylated	6	12	

* Significant at *P*< 0.05.

, we found concordant hypermethylation of *MGMT* and *CDH1* promoters (0.001), and *MGMT* and *RAR-β* promoters (0.0421). Similarly, hypermethylation of *CDH1* and *RAR-β* promoters (0.0005) was also found. However, we found no correlation between the methylation status of *MGMT* and *SYK* promoters, *CDH1* and *SYK* promoters or *RAR-β* and *SYK* promoters. There was no significant correlation between the DNA methylation status and clinical/pathological parameters with respect to age, tumour size and pathological grading.

### RT–PCR for RAR-β, RAR-β2, MGMT and SYK gene expression study

To examine the biological role of promoter hypermethylation in GCTs of ovaries, we assessed the levels of gene expression by semiquantitative RT–PCR in tumours with known methylation status in *RAR-β* gene. Both *RAR-β* and *RAR-β2* were found to have negative expression of mRNA in seven patients with OC showing promoter hypermethylation in *RAR-β* gene. Another patient was essentially negative for *RAR-β2* mRNA, although it shows aberrant methylation in the *RAR-β* promoter region. All other patients of OC patients showed normal expression of both *RAR-β* and *RAR-β2* and were not methylated (Table 2). The data suggest that promoter hypermethylation leading to gene silencing may affect a variety of key pathways in germ cell tumorigenesis. Aberrant promoter methylation changes that occur in cancer are associated with transcriptional repression and loss of function of the gene by interrupting the binding of proteins involved in the transcription activator complex. Our gene expression analysis by RT–PCR demonstrated that all tumours that showed methylation of *RAR-β* genes also showed downregulation of mRNA levels in the methylated tumours. Thus in these cases, promoter hypermethylation is one mechanism whereby gene expression can be deregulated in GCTs. We also assessed the levels of gene expression in *MGMT* and *SYK* genes by semiquantitative RT–PCRs in these patients. Only three patients showed negative expression of mRNA in these genes. Two patients showed promoter hypermethylation in all four tumour suppressor genes and negative expression of mRNA in *RARβ*, *MGMT* and *SYK.*

## DISCUSSION

Monosomy 22 and trisomy 14 appear to be emerging as nonrandom chromosome abnormalities in this type of tumours, although it is also associated with other complex chromosome abnormalities. In light of the data, the present finding of monosomy 22 as the sole chromosome change in these tumours suggests that this karyotypic change, possibly followed by the acquisition of an extra chromosome 14 (trisomy), may be nonrandom but an early, nonobligatory event of the tumorigenesis of GCTs of the ovary (Dal Cin *et al*, 1997; Speleman *et al*, 1997, Van den Berghe *et al*, 1999). The loss of fidelity in the inherent order of replication of allelic pairs during cell division provides a source for generating mutations involving genetic (aneuploidy) and epigenetic (gene silencing or allelic inactivation) events required for the generation and establishment of malignant phenotype (Duesberg *et al*, 2000). As reported in previous studies and in the present findings, monosomy of chromosome 22 and trisomy 14 provide a prognostic factor for the disease (Speleman *et al*, 1997). This emphasizes the association between *MGMT*, *CDH1*, *SYK* and *RARβ* methylation and aneuploidy. Genome-wide hypomethylation due to reduced levels of DNA methyl transferase, Dnmt 1 may lead to the loss or gain of certain chromosomes, that is, monosomy 22 and/or trisomy 14 as seen in the present study. Therefore, altered methylation results in chromosomal instability and hence increased risk of developing cancers. Instability of chromosomes is of such fundamental importance to the development of human or animal cancers that possibly there may be a relationship between DNA methylation status and chromosome loss/gain in somatic cells (Eden *et al*, 2003, Gandet *et al*, 2003).

DNA methylation is an inheritable epigenetic change in human cancers and the transcriptional silencing by hypermethylation of CpG islands in the promoter region is becoming more recognised as a common mechanism for the inactivation of various tumour suppressor genes and also affects a number of molecular pathways in human cancer. About 50% of human genes have unmethylated CpG clusters at their 5′-regulatory sequences. Aberrant methylation or hypermethylation of CpG islands in the promoters is associated with transcriptional inactivation and silencing of tumour suppressor genes. DNA replication errors have been found in ovarian GCTs (Suzuki *et al*, 2000). These findings further suggest that DNA mismatch repair deficiency may also contribute to the pathogenesis of ovarian cancer, and this deficiency may be an early event in the development and progression of the disease. Studies on methylation of specific genes known to play important roles in tumour development have contributed greatly to our current understanding of ovarian cancer. We found hypermethylation in *MGMT* gene in 32.5% cases of OC (14 out of 43). There are reports in the literature regarding the promoter hypermethylation and gene silencing of *MGMT* in many cancers (Esteller *et al*, 2000; Rosas *et al*, 2001; Bae *et al*, 2002). We found hypermethylation in *E-cadherin gene* (*CDH1*) in 20.9% OCs (9 out of 43). Hypermethylation in *CDH1* gene and its reduced expression in gut, liver, prostate and breast cancers, which could be due to the disruption of intercellular adhesion and impairment of *β*-catenin mediated transactivation of cadherin–catenin complex (Graff *et al*, 1995; Sadot *et al*, 1998; Tamura *et al*, 2000; Corn *et al*, 2001; Oue *et al*, 2002), has been reported. DNA methylation in the *RAR-β* promoter region has been found in 18.6% ovarian tumours (8 out of 43). Methylation in the promoter region of *RAR-β* gene is frequently found to be associated with downregulation/loss of its expression in tissues affected with gastric carcinoma and in gastric cell lines, head and neck cancers, breast cancer and non-small-cell lung carcinoma (Xu *et al*, 1994, 1997; Hayashi *et al*, 2001; Oue *et al*, 2002). DNA hypermethylation in *SYK* gene has been found in 16.3% ovarian tumours (7 out of 43). DNA methylation and loss of *SYK* expression has been reported in breast cancer (Coopman *et al*, 2000; Yuan *et al*, 2001). It could be associated with DNA hypermethylation mediated by methyl-CpG binding proteins that bind to methylated cytosines and forms a repressive and inactive complex of proteins that helps in repressing the transcription including that of HDACs and are gene silencing (Wade *et al*, 1999). Similar type of results have been reported for hMLH1 gene in primary gastric cancers and endometrial cancers (Fleisher *et al*, 1999; Leung *et al*, 1999). Several genes including tumour suppressor genes and DNA repair genes such as *hMLH1*, *RB1*, *VHL*, *p15*, *p16*, *RASSF1A*, *MGMT* and *BRCA1* in human cancers (Herman *et al*, 1994, 1996, 1998; Merlo *et al*, 1995; Esteller *et al*, 1999a; Baldwin *et al*, 2000; Gras *et al*, 2001; Nakayama *et al*, 2001; Yoon *et al*, 2001) were shown to be epigenetically inactivated by DNA methylation in tumours. Hypermethylation of CpG islands of another tumour suppressor gene *RASSF1A* has been reported in ovarian and renal cell carcinoma (Yoon *et al*, 2001). One of the major mechanisms of tumour progression is thought to be the inactivation of tumour suppressor genes. This inactivation can be induced by mechanisms such as chromosomal deletion and loss of function mutation in the coding region of genes or by epigenetic alteration in the form of methylation of promoter regions. We found that 15 of the MSI-positive tumours had hypermethylation of either *p16*, *BRCA1* or *RASSF1A* gene, whereas none of the MSI-negative tumours (MSS) demonstrated the promoter hypermethylation and this could be due to the epigenetic inactivation of either *p16*, *BRCA1* or *RASSF1A* genes (unpublished data). While it is possible that most of the methylation events we observed contribute in some way to the carcinogenic process, it is unlikely that all the methylated loci play a direct role in ovarian tumorigenesis. Rather, methylation of only certain classes of genes, such as tumour suppressor genes may be crucial to cancer progression.

We found concordant hypermethylation in promoters of *MGMT* and *CDH1*, *MGMT* and *RAR-β*, *CDH1* and *RAR-β*. Retinoic acid (RA) has profound regulatory effects in the control of many biological processes such as development, immunomodulation, differentiation, proliferation, reproduction and apoptosis (Lotan, 1980). The regulation of cell growth and differentiation of normal, premalignant and malignant cells by retinoids is thought to result from the direct and indirect effects of retinoids on gene expression. These effects are mediated by the nuclear receptors, including RAR-*β*2 located at 3p24. Retinoic acid receptor beta has four alternative splicing forms and the *β*-2 form appears to possess tumour-antagonizing activity at least in some cancers. There are no reports of the mutations in the RAR-*β* gene, but it undergoes epigenetic inactivation by promoter methylation in tumours of different origin. Retinoic acid receptor beta underwent DNA promoter region methylation in significant percent in several other tumours such as lung (Virmani *et al*, 2000), cervical (Yang *et al*, 2001) bladder (Maruyama *et al*, 2001), and prostate (Maruyama *et al*, 2002). Squamous cell carcinomas and premalignant dysplasias demonstrate a specific downmodulation of RAR-*β* that can be restored by systemic retinoid therapy (Lotan, 1995). It has also been reported that RA induces upregulation of *CDH1* expression and the morphological change of fibroblastoid to epithelioid growth of human pancreatic cancer cell line SUIT-2 (Jimi *et al*, 1998). Promoter hypermethylation in three genes *MGMT*, *CDH1* and *RAR-β* together in seven cases of OC, which also show loss of expression of both *RAR-β* and *RAR-β2*, may be due to the involvement of the signalling pathway mediated by *β*-catenin and other factors in the tumorigenic process in these OCs. It has already been reported that the reduced expression of *E-cadherin* gene and its hypermethylation (Tamura *et al*, 2000), and the reduced expression of *RAR-β* due to the promoter hypermethylation may be associated with the reduced expression of *CDH1*. *E-cadherin* is involved in a signalling pathway mediated by *β*-catenin and lymphocyte enhancer factor and T cell transcription factors and can, therefore, explain the concordant hypermethylation in the promoter regions of these three genes. Promoter hypermethylation of *RAR-β2* may block or interfere with the retinoid signalling pathways in OC. Deregulation of this pathway results in constant activation of *β*-catenin, lymphocyte enhancer factor and T cell factor target genes, including c-*myc* and *cyclin D1*. For the cyclin-dependent kinase inhibitor *p16*, the silencing of the gene mediated by promoter hypermethylation seems to be an early event in the development and progression of the tumorigenic process in ovarian cancers. CpG methylation patterns are replicated together with DNA replication during the S phase (Stein *et al*, 1982), and the altered transcriptional regulation via aberrant promoter methylation could play an important and significant role in the tumorigenic process of these ovarian cancers. Our results suggest promoter methylation of tumour suppressor genes such as *MGMT*, *CDH1* and *SYK.* We also analysed the relationship between promoter methylation status and basal expression levels of *RAR-β*, *RAR-β2*, *MGMT* and *SYK* in GCTs of ovaries. Our data clearly show that methylation in the promoter region of *RAR-β* gene is correlated with downregulation/loss of *RAR-β2* gene expression of GCTs of ovaries. Therefore, it might be the only mechanism responsible for loss of or downregulation of *RAR-β* gene expression in this type of cancer and plays a critical role in the tumour growth and development, which can cause disruption in the retinoid signalling pathway, the metastasis-related process and DNA repair processes in OC. Similarly, out of 14 patients who show promoter hypermethylation in *MGMT*, three show loss of expression while out of 7 patients who show promoter hypermethylation in *SYK* gene, only three were found to have loss of expression. It is clear from these results that it is not always true for all the tumour suppressor genes to show a positive correlation between promoter hypermethylation and loss of expression in cancers.

There exist two different pathways that can contribute to the development of cancers; genome-wide hypomethylation may lead to the loss of chromosomes thus leading to chromosomal instability, whereas promoter methylation in certain tumour suppressor genes, which is responsible for gene silencing, can lead to the development of cancers in somatic cells. Therefore, the balance in DNA methylation is very important, and alteration in these may be protective in one pathway but deleterious in the other. It can therefore be concluded that promoter hypermethylation of *MGMT*, *CDH1*, *RAR-β* and *SYK* genes, and loss of expression of *RAR-β* and *RAR-β2* in ovarian cancers (GCTs) is relatively common and this may also be useful as a tumour marker for early diagnosis and subsequent disease monitoring. Hence, these epigenetic signatures could play a decisive role in designing treatment options for this category of ovarian cancer.

## References

[bib1] Bae SI, Lee HS, Kim SH, Kim WH (2002) Inactivation of O6-methylguanine-DNA methyltransferase by promoter CpG island hypermethylation in gastric cancers. Br J Cancer Res 86: 1888–1892 10.1038/sj.bjc.6600372 PMC2375420 12085181

[bib2] Baldwin RL, Nemeth E, Tran H, Shvartsman H, Cass I, Narod S, Karlan BY (2000) BRCA1 promoter region hypermethylation in ovarian carcinoma: a population-based study. Cancer Res 60: 5329–5333 11034065

[bib3] Chambon P (1996) A decade of molecular biology of retinoic acid receptors. FASEM J 10: 940–954 8801176

[bib4] Chu DH, Morita CT, Weiss A (1998) The Syk family of protein tyrosine kinases in T-cell activation and development. Immunol Rev 165: 167–180 9850860 10.1111/j.1600-065x.1998.tb01238.x

[bib5] Coopman PJ, Do MT, Barth M, Bowden ET, Hayes AJ, Basyuk E, Blancato JK, Vezza PR, McLeskey SW, Mangeat PH, Mueller SC (2000) The Syk tyrosine kinase suppresses malignant growth of human breast cancer cells. Nature (London) 406: 742–747 10963601 10.1038/35021086

[bib6] Corn PG, Heath EI, Heitmiller R, Fogt F, Forastiere AA, Herman JG, Wu TT (2001) Frequent hypermethylation of the 5′ CpG island of E-Cadherin in esophageal adenocarcinoma. Clin Cancer Res 7: 2765–2769 11555590

[bib7] Dal Cin P, Qi H, Pauwels P, Backx C, Van den Berghe H (1997) Monosomy 22 in a fibrothecoma. Cancer Genet Cytogenet 99: 129–131 9398868 10.1016/s0165-4608(97)00210-0

[bib8] de The H, Vivanco-Ruiz MM, Tiollais P, Stunnenberg H, Dejean A (1990) Identification of a retinoic acid response element in the retinoic acid receptor b gene. Nature (London) 343: 177–180 2153268 10.1038/343177a0

[bib9] Duesberg P, Li R, Rasmick D, Rausch C, Willer A, Kraemer A, Yerganian G, Hehlman R (2000) Aneuploidy precedes and segregates with chemical carcinogenesis. Cancer Genet Cytogenet 119: 83–93 10867141 10.1016/s0165-4608(99)00236-8

[bib10] Eden A, Gandet F, Waghmare A, Jaenisch R (2003) Chromosomal instability and tumour promoted by DNA hypomethylation. Science 300: 455 12702868 10.1126/science.1083557

[bib11] Esteller M, Catasus L, Matias-Guiu X, Mutter GL, Prat J, Baylin SB, Herman JG (1999a) hMLH1 promoter hypermethylation is an early event in human endometrial tumorigenesis. Am J Pathol 155: 1767–1772 10550333 10.1016/S0002-9440(10)65492-2 PMC1866976

[bib12] Esteller M, Hamilton SR, Burger PC, Baylin SB, Herman JG (1999b) Inactivation of the DNA repair gene *O6-methylguanine-DNA methyltransferase* by promoter hypermethylation is a common event in primary human neoplasia. Cancer Res 59: 793–797 10029064

[bib13] Esteller M, Toyota M, Sanchez-Cespedes M, Capella G, Peinado MA, Watkins DN, Issa JP, Sidransky D, Baylin SB, Herman JG (2000) Inactivation of the DNA repair gene *O*^6^-*Methylguanine-DNA Methyltransferase* by promoter hypermethylation is associated with G to A mutations in *K-ras* in colorectal tumorigenesis. Cancer Res 60: 2368–2371 10811111

[bib14] Evan MF, McDicken IW, Herrington CS (1999) Numerical abnormalities of chromosomes 1, 11, 17, and X are associated with stromal invasion in serous and mucinous epithelial ovarian tumours. J Pathol 189: 53–59 10451488 10.1002/(SICI)1096-9896(199909)189:1<53::AID-PATH393>3.0.CO;2-U

[bib15] Fleisher AS, Esteller M, Wang S, Tamura G, Suzuki H, Yin J, Zou TT, Abraham JM, Kong D, Smolinski KN, Shi YQ, Rhyu MG, Powell SM, James SP, Wilson KT, Herman JG, Meltzer SJ (1999) Hypermethylation of the hMLH1 gene promoter in human gastric cancers with microsatellite instability. Cancer Res 59: 1090–1095 10070967

[bib16] Gandet F, Hodgson JG, Eden A, Jackson-Grusby L, Dausman J, Gray JW, Leonhardt H, Jaenisch R (2003) Induction of tumours in mice by genomic hypomethylation. Science 300: 489–492 12702876 10.1126/science.1083558

[bib17] Garcia del Muro X, Torregrosa A, Munoz J, Castellsague X, Condom E, Vigues F, Arance A, Fabra A, Germa JR (2000) Prognostic value of the expression of E-cadherin and *β*-catenin in bladder cancer. Eur J Cancer 36: 357–362 10708937 10.1016/s0959-8049(99)00262-2

[bib18] Gebert JF, Moghal N, Frangioni JV, Sugerbaker DJ, Neel BG (1991) High frequency of retinoic acid receptor *β* abnormalities in human lung cancer. Oncogene 6: 1859–1868 1717924

[bib19] Graff JR, Herman JG, Lapidus RG, Chopra H, Xu R, Jarrard DF, Isaacs WB, Pitha PM, Davidson NE, Baylin SB (1995) E-cadherin expression is silenced by DNA hypermethylation in human breast and prostate carcinomas. Cancer Res 55: 5195–5199 7585573

[bib20] Gras E, Catasus L, Arguelles R, Moreno-Bueno G, Palacios J, Gamallo C, Matias-Guiu X, Prat J (2001) Microsatellite instability, MLH-1 promoter hypermethylation, and frame shift mutations at coding mononucleotide repeat microsatellites in ovarian tumours. Cancer 92: 2829–2836 11753956 10.1002/1097-0142(20011201)92:11<2829::aid-cncr10094>3.0.co;2-3

[bib21] Hayashi K, Yokozaki H, Naka K, Yasui W, Lotan R, Tahara E (2001) Overexpression of retinoic acid receptor induces growth arrest and apoptosis in oral cancer cell lines. Jpn J Cancer Res 92: 42–50 11173543 10.1111/j.1349-7006.2001.tb01046.x PMC5926583

[bib22] Heimann R, Lan F, McBride R, Hellman S (2000) Separating favourable from unfavourable prognostic markers in breast cancer: the role of E-cadherin. Cancer Res 60: 298–304 10667580

[bib23] Herman JG, Jen J, Merlo A, Baylin SB (1996) Hypermethylation-associated inactivation indicates a tumor suppressor role for p15INK4B. Cancer Res 56: 722–727 8631003

[bib24] Herman JG, Latif F, Weng Y, Lerman LI, Zbar B, Lin S, Samid D, Duan DS, Gnarra JR, Linechan WM (1994) Silencing of the VHL tumor-suppressor gene by DNA methylation in renal carcinoma. Proc Natl Acad Sci USA 91: 9700–9704 7937876 10.1073/pnas.91.21.9700 PMC44884

[bib25] Herman JG, Umar A, Polyak K, Graff JR, Ahuja N, Issa JP, Markowitz S, Willson JK, Hamilton SR, Kinzler KW, Kane MF, Kolodner RD, Vogelstein B, Kunkel TA, Baylin SB (1998) Incidence and functional consequences of hMLH1 promoter hypermethylation in colorectal carcinoma. Proc Natl Acad Sci USA 95: 6870–6875 9618505 10.1073/pnas.95.12.6870 PMC22665

[bib26] Jimi S, Shono T, Tanaka M, Kono A, Yamada Y, Sudo K, Kuwano M (1998) Effect of retinoic acid on morphological changes of human pancreatic cancer cells on collagen gels: a possible association with the metastatic potentials. Oncol Res 10: 7–14 9613452

[bib27] Leung SY, Yuen ST, Chung LP, Chu KM, Chan AS, Ho JC (1999) hMLH1 promoter methylation and lack of hMLH1 expression in sporadic gastric carcinomas with high-frequency microsatellite instability. Cancer Res 59: 159–164 9892201

[bib28] Lotan R (1980) Effects of vitamin A and its analogs (retinoids) on normal and neoplastic cells. Biochim Biophys Acta 605: 33–91 6989400 10.1016/0304-419x(80)90021-9

[bib29] Lotan R (1995) Retinoids and apoptosis: implications for cancer chemoprevention and therapy. J Natl Cancer Inst 87: 1655–1657 7473809 10.1093/jnci/87.22.1655

[bib30] Lotan R, Xu C, Lippman SM, Ro JY, Lee JS, Lee JJ, Hong WK (1995) Suppression of retinoic acid receptor *β* in premalignant oral lesions and its up-regulation by isotretinoin. N Engl J Med 332: 1405–1410 7723796 10.1056/NEJM199505253322103

[bib31] Maruyama R, Toyooka S, Toyooka KO, Harada K, Virmani AK, Zochbauer-Muller S, Farinas AJ, Vakar-Lopez F, Minna JD, Sagalowsky A, Czerniak B, Gazdar AF (2001) Aberrant promoter methylation profile of bladder cancer and its relationship to clinicopathological features. Cancer Res 61: 8659–8663 11751381

[bib32] Maruyama R, Toyooka S, Toyooka KO, Virmani AK, Zochbauer-Muller S, Farinas AJ, Minna JD, McConnell J, Frenkel EP, Gazdar AF (2002) Aberrant promoter methylation profile of prostate cancers and its relationship to clinicopathological features. Clin Cancer Res 8: 514–519 11839671

[bib33] Merlo A, Herman JG, Mao L, Lee DJ, Gabreilson E, Burger PC, Baylin SB, Sidransky D (1995) 5′-CpG island methylation is associated with transcriptional silencing of the tumour suppressor p16/CDKN2/MTS1 in human cancers [see comments]. Nat Med 1: 686–692 7585152 10.1038/nm0795-686

[bib34] Nakayama K, Takebayashi Y, Namiki T, Tamahashi N, Nakayama S, Uchida T, Miyazaki K, Fukumoto M (2001) Comprehensive allelotype study of ovarian tumours of low malignant potential: potential differences in pathways between tumours with and without genetic predisposition to invasive carcinoma. Int J Cancer 94: 605–609 11745452 10.1002/ijc.1499

[bib35] Oue N, Motoshita J, Yokozaki H, Hayashi A, Tahara E, Taniyama K, Matsusaki K, Yasui W (2002) Distinct promoter hypermethylation of p16^INK4a^, CDH1 and RAR-beta in intestinal, diffuse-adherant, and diffuse-scattered type gastric carcinomas. J Pathol 198: 55–59 12210063 10.1002/path.1170

[bib36] Rosas SL, Koch W, da Costa Carvalho MG, Wu L, Califano J, Westra W, Jen J, Sidransky G (2001) Promoter hypermethylation patterns of *p16*, O6-methylguanine-DNA-methyltransferase, and death-associated protein kinase in tumors and saliva of head and neck cancer patients. Cancer Res 61: 939–942 11221887

[bib37] Russel P, Bannatyne P (1989) Surgical Pathology of Ovaries. London: Churchill Livingstone

[bib38] Sadot E, Simcha I, Shtutman M, Ben-Ze'ev A, Geiger B (1998) Inhibition of *β*-catenin-mediated transactivation by cadherin derivatives. Proc Natl Acad Sci USA 95: 15339–15344 9860970 10.1073/pnas.95.26.15339 PMC28044

[bib39] Sandberg AA (1991) Chromosome abnormalities in human cancer and leukaemia. Mutat Res 247: 231–240 2011141 10.1016/0027-5107(91)90019-k

[bib40] Seewaldt VL, Johnson BS, Parker MB, Collins SJ, Swisshelm K (1995) Expression of retinoic acid receptor-b mediates retinoic acid-induced growth arrest and apoptosis in breast cancer cells. Cell Growth Differ 6: 1077–1088 8519684

[bib41] Speleman F, Dermaut B, De Potter CR, Van Gele M, Van Roy N, De Paepe A, Laureys G (1997) Monosomy 22 in a mixed germ cell-sex cord-stromal tumor of the ovary. Genes Chromosomes Cancer 19: 192–194 9219001

[bib42] Stein R, Gruenbaum Y, Pollack Y, Rajin A, Cedar H (1982) Clonal inheritance of the pattern of DNA methylation in mouse cells. Proc Natl Acad Sci USA 79: 61–65 6459581 10.1073/pnas.79.1.61 PMC345661

[bib43] Suzuki M, Ohwada M, Saga Y, Ochiai K, Sato I (2000) DNA replication error is frequent in ovarian granulosa cell tumors. Cancer Genet Cytogenet 122: 55–58 11104034 10.1016/s0165-4608(00)00269-7

[bib44] Takeichi M (1995) Morphogenetic roles of classic cadherins. Curr Opin Cell Biol 7: 619–627 8573335 10.1016/0955-0674(95)80102-2

[bib45] Tamura G, Yin J, Wang S, Fleisher AS, Zou T, Abraham JM, Kong D, Smolinski KN, Wilson KT, James SP, Silverberg SG, Nishizuka S, Terashima M, Motoyama T, Meltzer SJ (2000) E-cadherin gene promoter hypermethylation in primary human gastric carcinomas. J Natl Cancer Inst (Bethesda) 92: 569–573 10.1093/jnci/92.7.569 10749913

[bib46] Van den Berghe I, Dal Cin P, De Groef K, Michielssen P, Van den Berghe H (1999) Monosomy 22 and trisomy 14 may be early events in the tumorigenesis of adult granulosa cell tumor. Cancer Genet Cytogenet 112: 46–48 10432935 10.1016/s0165-4608(98)00249-0

[bib47] Virmani AK, Rathi A, Zochbauer-Muller S, Sacchi N, Fukuyama Y, Bryant D, Maitra A, Heda S, Fong KM, Thunnissen F, Minna JD, Gazdar AF (2000) Promoter methylation and silencing of the retinoic acid receptor-beta gene in lung carcinomas. J Natl Cancer Inst 92: 1303–1307 10944551 10.1093/jnci/92.16.1303

[bib48] Wade PA, Gegonne A, Jones PL, Ballester E, Aubry F, Wolffe AP (1999) Mi-2 complex couples DNA methylation to chromatin remodelling and histone deacetylation. Nat Genet 23: 62–66 10471500 10.1038/12664

[bib49] Xu X-C, Ro JY, Lee JS, Shin DM, Hong YK, Lotan R (1994) Differential expression of nuclear retinoid receptors receptors in normal, premalignant and malignant head and neck tissues. Cancer Res 54: 3580–3587 8012985

[bib50] Xu X-C, Sneige N, Liu X, Nandagiri R, Lee JJ, Lukmanji F, Hortobagyi G, Lippman SM, Dhingra K, Lotan R (1997) Progressive decrease in nuclear retinoic acid receptor b messenger RNA levels during breast carcinogenesis. Cancer Res 57: 4992–4996 9371489

[bib51] Yang Q, Mori I, Shan L, Nakamura M, Nakamura Y, Utsunomiya H, Yoshimura G, Suzuma T, Tamaki T, Umemura T, Sakurai T, Kakudo K (2001) Biallelic inactivation of retinoic acid receptor beta2 gene by epigenetic change in breast cancer. Am J Pathol 158: 299–303 11141504 10.1016/s0002-9440(10)63969-7 PMC1850266

[bib52] Yoon JH, Dammann R, Pfeifer GP (2001) Hypermethylation of the CpG islands of the RASSF1A gene in ovarian and renal cell carcinomas. Int J Cancer 94: 212–217 11668500 10.1002/ijc.1466

[bib53] Yoshiura K, Kanai Y, Ochiai A, Shimoyama Y, Sugimura T, Hirohashi S (1995) Silencing of the E-cadherin invasion-suppressor gene by CpG methylation in human carcinomas. Proc Natl Acad Sci USA 92: 7416–7419 7543680 10.1073/pnas.92.16.7416 PMC41350

[bib54] Yuan Y, Mendez R, Sahin A, Dai JL (2001) Hypermethylation leads to silencing of the *SYK* gene in human breast cancer. Cancer Res 61: 5558–5561 11454707

[bib55] Zheng Z, Pan J, Chu B, wong YC, Cheung AL, Tsao SW (1999) Down-regulation and abnormal expression of E-cadherin and *β*-catenin in nasopharyngeal carcinoma: close association with advanced disease stage and lymph node metastasis. Hum Pathol 30: 458–466 10208469 10.1016/s0046-8177(99)90123-5

